# Patient Satisfaction in Out-patient Services at a Tertiary Care Center: A Descriptive Cross-sectional Study

**DOI:** 10.31729/jnma.4917

**Published:** 2020-05

**Authors:** Lisasha Poudel, Swechhya Baskota, Prajita Mali, Priza Pradhananga, Nabina Malla, Bibek Rajbhandari, Susmita Nepal

**Affiliations:** 1 Department of Public Health, Om Health Campus, Kathmandu, Nepal; 2 Department of Emergency, Nepal Police Hospital, Kathmandu, Nepal

**Keywords:** *Nepal*, *patient satisfaction*, *tertiary care center*

## Abstract

**Introduction:**

Patient satisfaction is an important and commonly used valid indicator for the measurement of service quality. Patient responses to healthcare services are one of the best ways to obtain information about patient views regarding the quality of healthcare. The main aim of the study was to find out the patient's satisfaction level in the tertiary care center.

**Methods:**

A descriptive cross-sectional study was conducted among 94 outpatients at a tertiary care center. Data were collected after obtaining ethical clearance from the institutional review committee. Patients were selected conveniently who visited any four of the major department. We collected demographic data and the patient satisfaction towards outpatient clinic experience was studied. We used the Patient Satisfaction Questionnaire-18 to assess patient satisfaction. Data were entered and analyzed in Statistical Package for the Social Sciences version 23. The mean score and the standard deviation were calculated.

**Results:**

Overall satisfaction was 74.78% with a mean value of 3.7394±0.40128. The highest satisfaction score was found in regards to the interpersonal manner of health personnel (4.2872±0.61561) followed by communication (3.9628±0.40982) and the lowest was seen in accessibility and convenience (3.2394±0.81478).

**Conclusions:**

The mean score and percentage of patient satisfaction were high in the hospital. However, the accessibility and availability of medical personnel were only a matter of concern.

## INTRODUCTION

Patient satisfaction is an important and commonly used valid indicator for the measurement of service quality.^1^ Patient responses to healthcare services are one of the best ways to obtain information about patient view regarding the quality of healthcare.^2^

Due to increasing emphasis on patients as consumers of medical services in the medical marketplace, patient satisfaction has emerged as a critical outcome of medical care.^1^ Patients perceptions of health care services seem to have been ignored by health care providers in developing countries.^3^ In Nepal, few studies of user perspectives of healthcare services have been undertaken. The quality factors that are pertinent to hospital clients include attitude, interpersonal, accessibility, technical skills of service personnel.^4^

The main aim of this study was to find the level of satisfaction among outpatients visiting four major departments regarding outpatient department services provided by tertiary care center.

## METHODS

A descriptive cross-sectional study was conducted among 94 outpatients in Bir Hospital, Kathmandu, Nepal after obtaining ethical clearance from the Institutional Review Committee (Ref no. 35). The data collection period was one month from June to July of 2019. Written consent was taken from the patients. Patients of 18 years old or above attending four major outpatient departments; surgery, medicine, urology, and dermatology were included in the study.

Patients who did not give consent, who were critically ill and admitted in an indoor or attending emergency were excluded. Prevalence i.e. 86% was taken from a study conducted in Nepal.^5^

The sample size was calculated using the following formula,

n= Z^2^ × (p × q)/e^2^

= (2.58)^2^ × (0.86) × (0.14)/(0.1)^2^

= 0.801/0.01

= 80.1

= 80

Where,

n= sample size
p= prevalence, 86%^5^
q= 1-p
= 1-0.86
= 0.14
e= margin of error, 10%
Z= 2.58 at 99% confidence interval.

Hence, the sample size calculated was 80. Taking non-response rate of 17%, the total sample size was 94. The sample of 94 were selected by using convenient sampling technique.

The tool used for data collection in the study was a semi-structured questionnaire with the Patient Satisfaction Questionnaire (PSQ-18) to assess patient satisfaction. It was designed to assess the patient satisfaction in seven fields: general satisfaction (3 and 17 items), technical quality (2,4,6 and 14 items), interpersonal manner (10 and 11 items), communication (1 and 13 items), financial aspects (5 and 7 items), time with the doctor (12 and 15 items) and access, availability/Convenience (8,9,16 and 18 items). PSQ-18 was translated in Nepali language and pretesting was done taking 10% of the total sample size. Cronbach alpha was calculated of the translated questionnaire, which was 0.79. During data collection, an exit interview was done.

Data were entered and analyzed in Statistical Package for the Social Sciences version 23. Analysis of the data was done by Statistical Package for Social Sciences version 23. Patient Satisfaction was determined by mean scores. Questions were scored on a five-point Likert scale (totally agree, agree, not sure, disagree, and totally disagree). PSQ-18 has nine positively worded items, which denote satisfaction related to health services received and nine negatively worded items which denote dissatisfaction related to health services received. In scoring instruction of PSQ-18, positively worded items were reversed and recorded in such a way that high scores reflect satisfaction with health care services received (Table 1).^1^

**Table 1:** Scoring items.

Items Numbers	Original Response Value	Scored Value
1, 2, 3, 5, 6, 8, 11,	1	5
15, 18	2	4
	3	3
	4	2
	5	1
4, 7, 9, 10, 12, 13,	1	1
14, 16, 17	2	2
	3	3
	4	4
	5	5

After item scoring, items within each scale were averaged together to create seven subscale scores. Based on a study conducted by Chakraborty et al. on patient satisfaction in an Urban Health Care Centre, scale score was done (Table 2).^6^

**Table 2:** Calculation of the level of satisfaction in terms of seven domains.

PSQ-18	Items	Maxi-mum Possible score	Maxim-um Mean	Level of satisf-action in percentage
General	3+	10	5	A
Satis-				
faction	17(A)			/10*100
Technical	2+4+6	20	5	B
quality	+14(B)			/20*100
Interpersonal	10+	10	5	C
manner	11(C)			/10*100
Communication	1+	10	5	D
	13(D)			/10*100
Financial aspects	5+7(E)	10	5	E
				/10*100
Time	12+15	10	5	F
with	(F)			
doctor				/10*100
Accessibility	8+9	20	5	G
and	+16+			/20*100
Convenience	18(G)			
Overall	All 18	90	5	OSAT
satis-	scales			
faction (OSAT)				/90*100

## RESULTS

The table shows the mean score and standard deviation of each domain of patient satisfaction. Hospital means satisfaction was the highest for interpersonal manner (4.2872), followed by communication (3.9628), technical quality (3.9468), financial aspect (3.7181), time spent with the doctor (3.6809), general satisfaction (3.6330) and least in accessibility and convenience (3.2394) (Table 3). Overall satisfaction was 74.78% with a mean value of 3.7394.

**Table 3:** Overall patient satisfaction scores towards tertiary care center (n=94).

Domains	Mean±S.D.	Level of Satisfaction (%)
General Satisfaction	3.6330±0.66468	72.66
Technical quality	3.9468±0.35140	78.93
Interpersonal Manner	4.2872±0.61561	85.74
Communication	3.9628±0.40982	79.25
Financial Aspect	3.7181±0.89670	74.36
Time spent with the doctor	3.6809±0.87021	73.61
Accessibility/Availability and Convenience	3.2394±0.81478	64.78
Overall Satisfaction (OSAT)	3.7394±0.40128	74.78

The mean age of the respondent was 44.09 years. Most of the respondents were female. Similarly, most of the respondents were married 77 (81.9%). The majority of the respondents were also Brahmin/Chhetri 47 (50%) followed by Janajati/Adhivasi 33 (35.1%), Dalit number 9 (9.6%), others 3 (3.2%) and Terai/Madhesi 2 (2.1%). The illiteracy rate of the respondents was 27 (28.7%). The majority of the respondents said that they were involved in homemakers (household) when asked about their occupation. Other than the household, the major occupation of the respondents was agriculture 18 (19.1%). The majority of the respondents had their monthly income of more than Nrs. 15000. Similarly, 37 (39.4%) were from rural areas and 57 (60.6%) were from urban areas at the public hospital. Each 25 (26.5%) patients were taken from the department of medicine, surgery, and urology while 14 (20.5%) patients were taken from the department of dermatology. About 89 (94.7%) of respondents were for follow up and 5 (5.3%) of them were new cases/first time visits. Forty-five (47.1%) patients were suffering from chronic illness and 49 (52.9%) patients were suffering from an acute illness (Table 4).

**Table 4:** Descriptive statistics of socio-demographic data (n=94).

Variables	n (%)
**Age (in years)**	
18-30	23 (24.5)
31-45	26 (27.7)
46-60	29 (30.9)
>61	16 (17.0)

	(Mean age: 44.09 years)
**Gender**	
Male	32 (34)
Female	62 (66)
**Marital status**	
Married	77 (81.9)
Single	17 (18.1)
**Ethnicity**	
Brahmain/Chhetri	47 (50.0)
Terai /Madhesi other castes	2 (2.1)
Janajati/Adivasi	33 (35.1)
Dalit	9 (9.6)
Others	3 (3.2)
**Educational status**	
Illiterate	27 (28.7)
Literate	67 (71.3)
**Occupation**	
Business	11 (11.7)
Agriculture	18 (19.1)
Government job	5 (5.3)
Private job	11 (11.7)
Student	5 (5.3)
Homemaker	40 (42.6)
Others	4 (4.3)
**Monthly income (Nrs)**	
<15000	43 (45.7)
>15000	51 (54.3)
**Residence**	
Rural	37 (39.4)
Urban	57 (60.6)
**Department**	
Medicine	25 (26.5)
Surgery	25 (26.5)
Urology	25 (26.5)
Dermatology	14 (20.5)
**Purpose of visit**	
Follow up	89 (94.7)
First-time visit/New case	5 (5.3)
**Type of illness**	
Chronic	45 (47.9)
Acute	49 (52.1)

Among subgroups, males were more satisfied than the female with accessibility and convenience whereas females were more satisfied with the financial aspect. Similarly, married patients were more satisfied than single patients with all seven domains. Literate people were more satisfied than illiterate people with financial aspects and accessible/convenience. But other than this domain, illiterate patients were more satisfied with general satisfaction, technical quality, interpersonal manner, communication, and time spent with the doctor. Urban residents were more satisfied than rural ones with technical quality, interpersonal manner, communication, financial aspect, and accessibility. Similarly, patients earning less than Nrs. 15000 as their monthly income were less satisfied than patients earning more than Nrs. 15000 in regard to all six domains. Patients who came for follow up were more satisfied than patients for new cases/first time visits with general satisfaction, financial aspect, and accessibility.

## DISCUSSION

As we know mismatch between patient expectation and the service received is related to decrease satisfaction, it is important to measure the level of patient satisfaction. So, determining patient perception and their level of satisfaction empowers them, which makes the health service more transparent and responsive to people`s needs.^7^One of the major components of quality health care is patient satisfaction and the research has also pointed out the clear link between patient outcome and patient satisfaction score.^6^

This study aims to find out the level of patient satisfaction at the tertiary care Centre situated in Kathmandu, Nepal. The findings show that the overall patient satisfaction was 74.78% with a mean score of 3.7394, which is lower than the study conducted in Nepal medical college teaching hospital that shows 86% of overall patient satisfaction.^5^ This might be due to the accessibility and availability of health care services provided by the hospital.

The mean score of the interpersonal manner of doctors was 4.2872, highest among seven domains of patient satisfaction. In the study, satisfaction level regards to interpersonal manner were higher when compared with the studies conducted in Patan Hospital and Tribhuvan University Teaching Hospital, Nepal.^8^,^9^ Most of the patients were satisfied with their doctor's attitude. Several doctors were presented in an examination room where several patients went inside at a time. Specialists discussed and examined the patients. This was one of the effective ways to save time and provide a treatment where specialists discussed among themselves.

About 35.22% of participants were dissatisfied about accessibility, availability, and convenience which contracts with the study conducted by Dafaalla M et al. that shows about only 20% of dissatisfaction in regards to accessibility and convenience.^10^

About 78.93% of the patients were satisfied with the medical equipment, doctor`s ability, and the diagnosis procedure. The vast majority of the patients referenced how the treatments were provided by well trained and experienced specialists. The only matter of concern was the accessibility and availability of doctors. Patients had to wait in a long queue to get the health services in the hospital. Regarding the financial aspect and time spent with doctors, the mean score and satisfaction percentage were quite good.

Having good communication improves the outcome of patient-doctor interaction. One of the most important components of good medical practice is good communication between patients and health care providers because it helps to solve the problem quickly and also establish trust between the physician and the patient. The satisfaction of patients regarding the communication skills of the doctor was high i.e. 79.25% with mean score 3.96 which contracts with the study conducted in Pakistan that shows a mean satisfaction score of 3.66.^11^ This indicates that there was a good doctor patient's relationship in the hospital.

Our study has some limitations. Firstly, a convenient sampling technique was done to select the sample population. Also, a tertiary care center was selected as our setting for data collection so, findings cannot be generalized concerning whole country settings.

## CONCLUSIONS

The mean score and percentage of patient satisfaction were high in the hospital. We can see a high satisfaction percentage in domains like interpersonal manner and communication skills of doctors towards their patients. However, the accessibility and availability of medical personnel were only a matter of concern. Lack of accessibility and availability of doctors on time was the main reason for dissatisfaction among dissatisfied patients.

## Conflicts of Interest:

**None.**
